# A cross sectional study investigating the association between exposure to food outlets and childhood obesity in Leeds, UK

**DOI:** 10.1186/s12966-014-0138-4

**Published:** 2014-12-06

**Authors:** Claire Griffiths, Anna Frearson, Adam Taylor, Duncan Radley, Carlton Cooke

**Affiliations:** Institute for Sport, Physical Activity and Leisure, Leeds Metropolitan University, Fairfax Hall, Headingley Campus, Leeds, LS6 3QS UK; The Office of the Director of Public Health Technorth, 9 Harrogate Road, Chapel Allerton, LS7 3NB UK; The Office of the Director of Public Health, NHS Leeds/Leeds City Council, 2nd Floor West - Civic Hall, Leeds, LS1 1UR UK

**Keywords:** Children, Obesity, Food environment

## Abstract

**Background:**

Current UK policy in relation to the influence of the ‘food environment’ on childhood obesity appears to be driven largely on assumptions or speculations because empirical evidence is lacking and findings from studies are inconsistent. The aim of this study was to investigate the number of food outlets and the proximity of food outlets in the same sample of children, without solely focusing on fast food.

**Methods:**

Cross sectional study over 3 years (n = 13,291 data aggregated). Body mass index (BMI) was calculated for each participant, overweight and obesity were defined as having a BMI >85^th^ (sBMI 1.04) and 95^th^ (sBMI 1.64) percentiles respectively (UK90 growth charts). Home and school neighbourhoods were defined as circular buffers with a 2 km Euclidean radius, centred on these locations. Commuting routes were calculated using the shortest straight line distance, with a 2 km buffer to capture varying routes. Data on food outlet locations was sourced from Leeds City Council covering the study area and mapped against postcode. Food outlets were categorised into three groups, supermarkets, takeaway and retail. Proximity to the nearest food outlet in the home and school environmental domain was also investigated. Age, gender, ethnicity and deprivation (IDACI) were included as covariates in all models.

**Results:**

There is no evidence of an association between the number of food outlets and childhood obesity in any of these environments; Home Q4 vs. Q1 OR = 1.11 (95% CI = 0.95-1.30); School Q4 vs. Q1 OR = 1.00 (95% CI 0.87 – 1.16); commute Q4 vs. Q1 OR = 0.1.00 (95% CI 0.83 – 1.20). Similarly there is no evidence of an association between the proximity to the nearest food outlet and childhood obesity in the home (OR = 0.77 [95% CI = 0.61 – 0.98]) or the school (OR = 1.01 [95% CI 0.84 – 1.23]) environment.

**Conclusions:**

This study provides little support for the notion that exposure to food outlets in the home, school and commuting neighbourhoods increase the risk of obesity in children. It seems that the evidence is not well placed to support Governmental interventions/recommendations currently being proposed and that policy makers should approach policies designed to limit food outlets with caution.

## Background

In the UK 1 in 3 children and young people (approximately 4.5 million) are overweight or obese [1]. The harmful effects of obesity are not only experienced by the individuals, through worsened health status, but also financially by society [2]. There is clear evidence of the significant direct and indirect costs that are associated with obesity. In the UK obesity-related illnesses costs the NHS an estimated £5.1 billion a year. As a result obesity is a cross government national priority in the UK with a national target to achieve a sustained downtrend in the level of excess weight in children by 2020 [3].

Of concern, contemporary obesity prevalence data provides little confidence that national or International childhood obesity targets can be met using existing approaches. Although governments have repeatedly attempted to address this issue, their approaches have been ineffective. There is now an urgent need to identify evidence-based policy to achieve national and International targets. The most comprehensive investigation into obesity and its causes [2] described obesity as a complex problem that requires action from individuals and society across multiple sectors. The food environment, broadly conceptualised to include any opportunity to obtain food, is one of the four major areas on the Obesity System Map developed by Foresight [2]. Perhaps as a result of this, much attention has recently focused on action to modify the food environment. Policy makers are beginning to engage [4] with the idea that food environments are a contributing factor to the obesity epidemic. Indeed public health professionals in the UK are encouraged to address the prevalence of fast food outlets in their area to support healthier lifestyles [4,5].

To date the primary focus has been food availability around schools. Changes to the distribution and density of fast food outlets around schools have been proposed in the UK [5–8], as part of health policy. However, the empirical foundation of such food availability based approaches, including the impact on excess weight gain, is still unclear due to an equivocal evidence base. Harris et al. [9] concluded that un-healthy food choices are ubiquitous and there is no association between stores selling these foods in close proximity to US schools and obesity rates. In contrast, other American studies have reported that fast food restaurants within close proximity to a school can significantly effect school obesity rates [10–12]. Other studies have found different associations between different types of food outlet [13] with convenience stores reducing the risk of obesity, grocery stores increasing the risk and no association with fast food outlets. A recent systematic review did not find any evidence to support policies aimed at regulating the food environment around schools [14]. Although over half of the associations between food outlets around schools and body weight reported in the review showed a positive relationship, only 19 of these were statistically significant [14]. Thus almost 75% of the 72 relationships were either negative or not statistically significant. These contrasting findings may in part be due to the inherent complexities in the methods, including definitions of food outlets, a focus solely on fast food and an over reliance on US based studies.

Less research has considered the food environment and obesity at the individual household level in children. However, findings are equally unclear. Some report positive associations between food outlet density (i.e. count) in a child’s neighbourhood and obesity [15,16] whilst others report no effect [17–19] or even an inverse relationship [20]. Further, research investigating the association between proximity (i.e. distance) of food outlets and childhood obesity report no significant associations between the proximity to the nearest fast food outlet and whether a child was obese or not [16,21]. Results from longitudinal studies, which offer stronger evidence on associations, are also inconclusive. While national level analysis of children measured over time reported a significant association between supermarket availability and lower BMI [22], others report that differential exposure to food outlets over time does not independently explain weight gain in children [19,23].

Although the association between food availability and childhood obesity is uncertain there does seem to be consistent associations between the number of food outlets, in particular fast food outlets, and deprivation [14,16,24,25], and there is support from the literature that obesity is closely associated with deprivation [1], although this suggested linear relationship has been questioned [26]. It is therefore likely that the areas with the highest prevalence of obesity are also likely to have the highest density of food outlets. This pattern suggests a plausible hypothesis that food availability inequalities are correlated with and may contribute to obesity inequalities. However, these are only observational data which therefore give no evidence of causation. Furthermore, most of this prior research has focused only on fast food and takeaway (varied definitions) availability and so does not allow for the possibility that other food outlet types (supermarkets, convenience stores etc.) may be patterned in a similar way. For example, Pearce et al. [25] demonstrated that outlets selling some healthy food (e.g. supermarkets) are patterned by deprivation in a similar way to fast food outlets in New Zealand, and similar results have been shown in the US [20,23]. It is possible that the areas with the highest prevalence of obesity are likely to have the highest density of all food outlet types, not just takeaways.

One of the ten recommendations of the Academy of Medical Royal Colleges’ 2013 report on obesity was that “Public Health England should, undertake an audit of local authority licensing and catering arrangements with the intention of developing formal recommendations on reducing the proximity of fast food outlets to schools, colleges, leisure centres and other places where children gather” [5]. Although the most recent briefing paper from PHE [4] supports this recommendation, it also states that there is *‘an unavoidable lack of evidence that can demonstrate a causal link between actions and outcomes*’. Furthermore the document highlights that *‘taking action on hot food takeaways is only part of the solution, as it does not address sweets and other high-calorie food that children can buy in shops near schools’.* It seems that current UK recommendations in relation to the influence of the ‘food environment’ are driven largely on assumptions or speculations because empirical, UK specific evidence is lacking. The current evidence base is not well placed to support the recommendations currently being proposed.

This study aims to investigate the association between childhood obesity and both the number of food outlets and proximity of food outlets to the child household, school and in commuting (between home and school) environments. As far as the authors are aware this is the first UK study to undertake this analysis, in the same sample of children, in the three different environments, using weight status as the outcome measure.

## Methods

### Study population

Data are from the Rugby League and Athletics development Scheme (RADs) which is a collaboration between Leeds City Council, Leeds Metropolitan University and the Education authority (formally Education Leeds). Ethical clearance was granted by the ethics committee of the Carnegie Faculty, Leeds Metropolitan University. Cross sectional data from RADs has been reported previously [26,27]. Only children living within the study area (Leeds boundary) and with a valid BMI (i.e. calculated from measured height and weight), postcode and reported ethnicity were included within this study. The final analyses were based on 13 291 participants from 37 secondary schools (for the school environment analysis 1 school was excluded and because since testing took place the school has closed down).

### Measures

All testing took place on school premises. Stature was measured to the nearest 0.1 cm using a floor-standing Leicester height measure (model 220) with children standing erect without shoes. Weight was measured to the nearest 0.01 kg using manually calibrated electronic scales (Tanita TBF-310; Tanita, Tokyo, Japan), without shoes. All measurements were taken by the same person (CG) between September and December of each measurement year (2005 n = 4727; 2006 N = 4480; 2007 N = 4084). Technical error of measurement and coefficient of variation demonstrate appropriate reliability [26,27].

#### Outcome measures

Body mass Index (BMI) was calculated for each participant as weight (kg)/height^2^ (m) and converted to a standard deviation score (sBMI) using the British 1990 growth reference charts (UK90) for BMI [28] to allow comparison while accounting for normal growth (age and gender). Children were also classified as overweight or obese on the basis of their sBMI score. The 85^th^ (sBMI = 1.04) and 95^th^ (sBMI = 1.64) centiles were used to define overweight and obesity respectively.

#### Individual measures

Age, gender, ethnicity (White-British and other), and deprivation scores (Income of Deprivation Affecting Children [IDACI, 2007]) were included as covariates. IDACI scores were assigned to the lower super-output area (LSOA) of each individual and school, as determined by postcode.

#### Exposure to food outlets

Participant’s home and school addresses were mapped by postcode using a geographic information system (MapInfo Professional). Home and school neighbourhoods were defined as circular buffers with a 2 km Euclidean (straight line) radius, centred on these locations. Accurate data on food outlet locations was sourced from Leeds City Council covering the study area during the time of data collection and again mapped by postcode. Food outlets were categorised into three groups, supermarkets, takeaways and retail (including petrol stations) according to the data base held by LCC. The original data base included takeaway outlets by cuisine (e.g. Indian, Chinese etc.) these were collapsed to one category for this analysis, for simplicity of reporting results. Petrol stations were also a separate category however, for this analysis they were included in the retail category, due to small numbers of such outlets. No food outlets were excluded from the data base provided by LCC. All outlets falling within the 2 km buffers were identified (supermarkets, takeaway and retail separately and total outlets). The straight line distance from each child’s home and school postcode centroid to the nearest food outlet was calculated using (Distance Calculator tool, MapInfo Professional).

Finally commuting routes (home to school) were calculated according to the shortest straight line distance using (Distance Calculator tool, MapInfo Professional). Only children that lived within 2km of their school were included (n = 7501, 55%) in this analysis as this was considered a plausible walking distance. To capture varying routes a 2 km buffer was placed around this ‘shortest distance’ and the number of food outlets falling within the 2 km buffer were identified.

### Statistical analysis

Simple Pearson’s correlation was used to assess the relationship between SES and the number of fast food outlets and proximity to the nearest outlet.

Analysis was performed at the individual level (n = 13291). Results published previously on the RADs cross sectional data [26] suggested that single-level models are sufficient for analysing these data in all cases. The additional complexity of multi-level modelling (MLM) to model variation at different levels (i.e. pupils nested within schools and pupils within geographic areas) did not identify any variance at level 2 (i.e. between schools or between geographic areas) in the data set. It was unlikely that the addition of the food environment variables would result in significant level 2 (i.e. school or geographic area) variances. However, comparison of the same MLM models with the addition of the food environment variables were considered and showed the same substantive overall conclusions. Therefore fixed effect regression models are reported for simplicity.

We used multiple linear regression models (β and 95% confidence intervals reported) to estimate associations between the food environment (supermarkets, takeaway, retail separately and total outlets) and sBMI. Logistic regression models (odds ratios (OR) and 95% confidence intervals reported) were used to estimate the associations when using overweight and obesity as the outcome in the following environments:

- Child Household
- School
- Commute from home to school

The number of outlets within each environment varied considerably e.g. number of takeaways within the school environment ranged from 9 – 95 (Table 1). This should be acknowledged when interpreting outputs from statistical models. The outcomes reported here assume that the outcome (i.e. log odds for being obese or sBMI) increase by the same amount (β) for one unit increase in the food environment variable i.e. an increase from 3 to 4 outlets will have the same effect on the outcome as an increase from 94 to 95 outlets, which seems unlikely. Therefore we modelled exposure to food outlets in all environments as quarters of counts of food outlets using dummy variables (least exposed = Q1 (reference category), most exposed = Q4). Sensitivity analysis using exposure to food outlets as a continuous variable and taking the square root of the number of food outlets variable showed substantively the same conclusions.

**Table 1:** **Results of logistic regression investigating the association between the number of food outlets and obesity in the three different environments after adjustment for covariates**

	**Home**		**School**		**Commute**	
**2 km retail**						
Q1	REF	P	REF	P	REF	P
Q2	1.02 [0.89:1.16]	0.83	0.95 [0.83:1.08]	0.39	0.98 [0.83:1.17]	0.85
Q3	1.14 [0.99:1.31]	0.06	0.95 [0.83:1.07]	0.41	1.12 [0.94:0.33]	0.20
Q4	1.11 [0.95:1.30]	0.19	0.98 [0.85:1.13]	0.79	0.99 [0.82:1.21]	0.95
Range	0 - 167		2 - 89		0 - 93	
**2 km takeaways**						
Q1	REF	P	REF	P	REF	P
Q2	0.95 [0.83:1.08]	0.45	0.97 [0.85:1.10]	0.64	1.06 [0.89:1.25]	0.54
Q3	1.12 [0.98:1.28]	0.11	0.93 [0.83:1.05]	0.25	0.99 [0.84:1.18]	0.97
Q4	1.05 [0.90:0.22]	0.53	0.97 [0.84:1.13]	0.70	0.97 [0.80:1.16]	0.71
Range	0 – 165		3 – 95		0 - 88	
**2 km supermarkets**						
Q1	REF	P	REF	P	REF	P
Q2	0.96 [0.85:1.08]	0.47	1.03 [0.89:1.19]	0.68	0.85 [0.72:0.99]	0.04
Q3	0.97 [0.84:1.10]	0.58	1.02 [0.89:1.17]	0.80	0.79 [0.64:0.96]	0.02
Q4	1.03 [0.87:1.20]	0.68	1.00 [0.87:1.13]	0.99	1.02 [0.87:1.20]	0.80
Range	0 – 28		0 – 14		0 – 15	
**2 km total outlets**						
Q1	REF	P	REF	P	REF	P
Q2	1.04 [0.91:1.18]	0.60	0.95 [0.83:1.08]	0.42	1.02 [0.85:1.21]	0.87
Q3	1.11 [0.97:1.27]	0.15	0.92 [0.81:1.04]	0.17	1.07 [0.91:1.27]	0.41
Q4	1.11 [0.95:1.30]	0.18	1.00 [0.87:1.16]	0.95	1.00 [0.83:1.20]	0.99

Values = OR (95% confidence intervals); All models control for gender, ethnicity and SES (IDACI).

A food outlet count model was fitted for each outcome (sBMI, overweight and obese, obese only) with the independent variables of gender, ethnicity, IDACI and number of food outlets (quarter of counts) in each of the three environments was calculated. The same model was run for all outcomes replacing the number of food outlets variable with the distance to the nearest food outlet to the home and school environment. Age was not included in the models as all children were from the same school year (age 11–12).

All analyses were performed in SPSS version 21.

## Results

### Sample characteristics

Table 2 shows descriptive statistics for the study sample in the three environments. There was an even gender split however, most children were White British (80%). The prevalence of obesity was slightly higher in boys (19.8%) compared to girls (17.5%).

**Table 2:** **Characteristics of participants and food exposure in the different environments**

	**Individual**	
	**n = 13291**	
	**Boys (50%)**	**Girls (50%)**
Age	11.59 [0.30]	11.57 [0.30]
Ethnicity (%White)	83.1	82.4
IDACI	0.25[0.19]	0.25 [0.20]
BMI	19.01 [3.49]	19.59 [3.76]
BMIsd	0.50 [1.22]	0.42 [ 1.22]
% Overweight + obese	33.7	31.9
% Obese	19.8	17.5

Values are mean [standard deviation] unless otherwise stated.

### Associations between number of food outlets and obesity

The range of all types of food outlets within all three environments was large (Table 1). Associations between the number of food outlets (supermarkets, takeaway, retail separately and total outlets) and the probability of being obese are shown in Table 1, for the three environments after adjustment for the covariates. Results for overweight and obesity as the outcome variable reported similar results (data not shown). Unadjusted models showed no association between the number of food outlets and obesity, in the school or commute environments. However, significant associations were observed in the home environment and all food outlet types (takeaway Q4 OR = 1.20 [1.06: 1.36]; supermarkets Q4 OR = 1.18 [1.05: 1.34]; retail Q4 OR = 1.23 [1.11: 1.34]). The adjusted models provide no evidence of a significant association between the number of food outlets and childhood obesity in home or school environment. In both models the odds ratios reported are very close to one (i.e. the outcome is equally likely for both groups) and not statistically significant (95% confidence intervals all cross 1) for each quarter count of food outlets compared to the least exposed group (Q1) for all types of outlet (individually and combined). The only significant associations are observed in the home – school commute environment and the number of supermarkets (Table 1). It seems that children who potentially pass more supermarkets (Q2 and 3) are less likely to be obese compared to the children in Q1.

Associations between sBMI and the number of food outlets after adjustment for gender, ethnicity and IDACI are shown in Table 3. There are no statistically significant associations between the number of food outlets and sBMI in the child household and commute environments (with the exception of Q2 supermarkets in the commute model β = −0.10 [−0.17: −0.02]). At the school level there were significant associations with the number of retail outlets the number of takeaways and total outlets. Interestingly these associations are all negative.

**Table 3:** **Results of linear regression investigation the association between sBMI and the number of food outlets in the three different environments after adjustment for covariates**

	**Home**		**School**		**Commute**	
**2 km retail**						
Q1	REF	P	REF	P	REF	P
Q2	0.01 [-0.05:0.07]	0.76	-0.07 [-0.13:-0.01]	0.03	-0.24 [-0.10:0.06]	0.57
Q3	0.05 [-0.02:0.12]	0.14	-0.09 [-0.16:-0.03]	0.00	0.01 [-0.07:0.09]	0.82
Q4	0.02 [-0.05:0.10]	0.57	-0.00 [-0.07:0.06]	0.90	-0.02 [-0.11:0.07]	0.62
**2 km takeaways**						
Q1	REF	P	REF	P	REF	P
Q2	0.00 [-0.06:0.06]	0.98	-0.03 [-0.09:0.03]	0.37	-0.02 [-0.10:0.06]	0.59
Q3	0.03 [-0.04:0.09]	0.39	-0.06 [-0.12:-0.01]	0.03	-0.06 [-0.01:0.02]	0.14
Q4	0.00 [-0.07:0.07]	0.98	0.02 [-0.06:0.09]	0.69	-0.04 [-0.13:0.04]	0.32
**2 km supermarkets**						
Q1	REF	P	REF	P	REF	P
Q2	-0.01 [-0.07:0.05]	0.70	0.03 [-0.04:0.10]	0.40	-0.10 [-0.17:-0.02]	0.01
Q3	0.00 [-0.06:0.07]	0.92	0.04 [-0.03:0.10]	0.28	-0.08 [-0.18:0.01]	0.07
Q4	0.01 [-0.07:0.08]	0.82	0.01 [-0.05:0.07]	0.75	-0.02 [-0.10:0.06]	0.61
**2 km total outlets**						
Q1	REF	P	REF	P	REF	P
Q2	0.04 [-0.02:0.12]	0.17	-0.02 [-0.08:0.04]	0.48	-0.05 [-0.13:0.04]	0.29
Q3	0.04 [-0.03:0.10]	0.30	-0.08 [-0.14:-0.02]	0.01	-0.01 [-0.08:0.07]	0.90
Q4	0.04 [-0.04:0.11]	0.36	0.01 [-0.06:0.08]	0.74	-0.05 [-0.14:0.03]	0.23

Values are β [95% confidence intervals]; All models control for gender, ethnicity and SES (IDACI).

### Associations between proximity of food outlets and obesity

Associations between the proximity of the nearest food outlet (supermarkets, takeaway, retail separately and total outlets) and all outcomes after adjustment for gender, ethnicity and IDACI are shown in Table 4 (unadjusted models were not statistically significant). The average distance between the nearest food outlet and the child’s home and school were 0.27 km and 0.37 km respectively. The models considered the specific types of outlet e.g. children whose nearest food outlet was a take away (n = 3467 25.4%), retail (n = 9738 71.3%) or supermarket (n = 451 3.3%) and all outlets combined. The only statistically significant outcome was the proximity to a retail outlet (OR 0.67 [0.50:0.90]) and total outlets (OR 0.77 [0.61:0.98]) and the probability of being obese. In both models the odds ratios are less than one, suggesting that as the distance to the nearest outlet increases (i.e. gets further away) the probability of being obese also decreases. The model for overweight and obesity showed similar results (data not shown). Table 4 provides no evidence of a significant association between distance to the nearest food outlet and sBMI.

**Table 4:** **Results from regression analysis investigating the relationship between proximity of food outlets to the home and school environments after adjustment for covariates**

		**Takeaway**		**Retail**		**Supermarket**		**All outlets**	
			**P**		**P**		**P**		**P**
**Child home**	% of children	25.4 (n=3467)		71.3 (n=9738)		3.3 (451)		100	
	Distance (km)^1^	0.24 [0.23]		0.27 [0.22]		0.31 [0.24]		0.27 [0.22]	
	BMI Obese^2^	0.90 [0.58:1.40]	0.64	0.67 [0.50:0.90]	0.01	2.15 [0.82:5.67]	0.12	0.77 [0.61:0.98]	0.03
	BMIsds^3^	-0.08 [-0.28:0.12]	0.44	-0.10 [-0.22:0.29]	0.13	0.34 [-0.45:0.51]	0.89	-0.09 [-0.19:0.02]	0.10
**School**	n of schools	8/36		26/36		2/36			
	Distance^1^	0.42 [0.44]		0.34 [0.13]		0.48 [0.10]		0.37 [0.25]	
	BMI Obese^2^	1.08 [0.88:1.33]	0.47	0.85 [0.54:1.30]	0.85	1.73 [0.19:15.68]	0.63	1.01 [0.84:1.23]	0.95
	BMIsds^3^	-0.15 [-0.12:0.09]	0.78	-0.021[-0.21:0.20]	0.95	-0.24 [-1.17:0.69]	0.62	-0.01 [-0.10:0.08]	0.81

^1^mean [standard deviation] ^2^results from logistic regression models values = odds ratio [95% confidence intervals]; ^3^results from linear regression models values = β [95% confidence intervals): All models control for gender, ethnicity and SES (IDACI).

### Deprivation and food outlets

There was a significant positive relationship between the number of food outlets and SES at the school (r = 0.62 p < 0.05) and child household (r = 0.17 p < 0.05) level i.e. schools in more deprived areas and children who live in more deprived areas have more food outlets within 2km. Similar relationships were also found for the number of takeaways (school r = 0.58 p < 0.05; child household r = 0.13 p < 0.05), retail outlets (school r = 0.64 p < 0.05; child household r = 0.21 p < 0.05) and supermarkets (school r = 0.53 p < 0.05; child household r = 0.05 p < 0.05).

Statistically significant negative relationships between SES and proximity to the nearest food outlet at the school (r = −0.14 p < 0.05) and child household (r = −0.29 p < 0.05) level were also observed i.e. schools in more deprived areas and children who live in more deprived areas are closer to a food outlet. Again similar relationships were found for the number of takeaways (school r = −0.33 p < 0.05; child household r = −0.25 p < 0.05), retail outlets (school r = −0.37 p < 0.05; child household r = −0.31 p < 0.05) and supermarkets (school r = −0.10 p < 0.05; child household r = −0.23 p < 0.05).

## Discussion

To our knowledge, this is the first study to investigate the association between food exposure, and childhood obesity at the household, school and commuting environments in a large sample of children in the UK. Our key finding was that there is no evidence of a positive association between the number of food outlets or the proximity to the nearest food outlet and childhood obesity in any of these environments, when controlling for SES. Of particular importance is that this was true for all types of food outlet including takeaways and fast food outlets. Although there were some significant associations with exposure to food outlets and sBMI these were border line and negative (Table 1) which are similar to those reported by Crawford et al. [17].

At the school level these data are in agreement with a recent systematic review [14] which concluded that currently there is no evidence to support policies aimed at regulating the food environment around schools. At the child household level our data are in agreement with current evidence [17–19,21,23,29] in relation to fast food exposure. Together these studies do not support the assumption or hypothesis that fast food exposure in the local (i.e. home) neighbourhood increases the risk of obesity once you take into account gender, ethnicity and SES. Finally the lack of any association between exposure to food outlets and obesity in the commuting environment is supported by data from the UK [30] New Zealand [31] and Australia [31].

There are two central points to consider when interpreting findings from studies examining the associations between the food environment and obesity. Firstly, a simple stratification is often applied to classify food outlets, such that fast food, takeaways and convenience stores are typically identified as ‘unhealthy’ while grocery stores and supermarkets are used as a proxy for ‘healthy’ food. This over simplified classification ignores the wide range of unhealthy foods available at most, if not all supermarkets and the healthy foods available at most takeaways. It must be remembered that ‘healthy’ and ‘unhealthy’ food can be purchased almost anywhere and this distinction is important. The importance of this was highlighted by a US study which demonstrated that despite being classified as ‘healthy’, adolescents purchasing a meal at subway ordered just as many calories as those purchasing a meal at McDonalds, which was classified as ‘unhealthy’ [32]. It seems that the ‘health halo’ [32,33] may falsely portray some outlets as healthier options even though they may not be. Perhaps the ‘healthiness’ of a food outlet should be measured by what consumers actually purchase or consume. This point was highlighted in a recent study [13] which demonstrated that the number of convenience stores (OR 0.94, 95% CI [0.87:1.00]) which are typically classified as unhealthy, with a 2km radius of a child’s school actually reduced the risk of obesity and the association with fast food outlets, also classified as unhealthy in many studies was not statistically significant. However, the number of grocery stores, which are typically classified as healthy, actually increased the risk of being obese (OR1.06, 95% CI [0.99:1.12]).

Secondly the relationship/association between exposure and consumption is poorly understood. The notion that the count/proximity of fast food outlets influence consumption has intuitive appeal. It is plausible that greater exposure would be associated with greater consumption within the home or school environment. Studies have demonstrated that children who consume fast food (compared to children who do not consume fast food) have higher energy intake and higher fat intakes [34–36]. However, few studies have investigated if exposure to food outlets at either the school or household level is associated with greater consumption.

Timperio et al. [31] demonstrated that the density of stores close to a child’s home was negatively associated with consuming takeaways, however this association was borderline (OR 0.98 [95% confidence intervals 0.96 – 0.99]). There was also no association between the availability of fast food along the home-school commuting route and consumption [31]. Similar findings have also been reported in adults [29,37]. In contrast, Fraser et al. [38] demonstrated that teenagers who are exposed to more fast food outlets near their homes are more likely to eat fast food (β = 0.61, p < 0.001) and that this in turn was associated with a higher sBMI (β =9.2, p <0.001). Forsyth et al. [39] reported similar findings in boys but not girls. Studies that have considered the school environment [39–42] found no evidence to support the hypothesis that less exposure to fast food or better access to supermarkets are related to higher diet quality or lower BMI in children.

In epidemiological terms, mere proximity to a store may no longer be a good index of exposure. This notion has been demonstrated recently in adults [43] where physical distance to food outlets was unrelated to obesity risk in an international comparison of Seattle (n = 1340) and Paris (n = 7131). It therefore seems reasonable to suggest that the geographical location of food outlets may be relatively unimportant and not associated to childhood obesity. Perhaps factors such as food actually purchased, the range or choice of food available, the size and quality of food outlets [19], advertising, and cost of food may be more important determinants of adiposity than simple measures of exposure.

It seems that children who live in more deprived areas or schools located in more deprived areas have more takeaways within their neighbourhood. This relationship was stronger at the school level compared to the child household level (where although it was statistically significant it is very weak). In addition, children who live in more deprived areas or schools located in more deprived areas are closer to a takeaway than children or schools in less deprived areas (although these relationships are statistically significant they are weak).

Similar to other studies, the findings of this research show that children and schools in more deprived areas have more fast food outlets than children and schools in less deprived areas. This is despite very different definitions for fast food outlets used in these studies. However, when the picture is broadened to include other types of food outlet, children living in more deprived areas also have greater access to food establishments that are not perceived to be obviously linked to obesity risk, including retail stores and supermarkets. These findings are consistent with data from New Zealand [25] and the US [20,23]. It is plausible that neighbourhoods which have many fast food/takeaways may also have many ‘other’ types of food outlets, thus diluting the exposure to fast food. This is a particularly important finding and suggests that focusing on one particular outlet type does not truly characterise a person’s food environment. To gain a true reflection of a food environment it is important that all types of food outlets are considered. A strong positive relationship between fast food outlets and deprivation has been documented in the UK [44] and there is support from the literature that obesity is closely associated with deprivation [4]. However, this association could be deceptive because few studies have investigated the relationship between deprivation and other types of food outlets. While these data support the fast food –deprivation relationship, they also suggest that children living in more deprived areas also have more exposure to supermarkets and retail outlets. Perhaps a more important research question would be to consider the association between actual food availability/food purchased and deprivation.

It is relatively well established that obesity is closely associated with deprivation [1], although the linear relationship has been questioned [26]. A recent study suggested that only 1 – 2% of the total effect of deprivation on obesity in children was explained by the availability of fast food and other unhealthy food outlets in the environment [15]. This is in part supported by our data which shows that although there seems to be more takeaways in more derived areas there is no evidence of an association between the number of takeaways or the proximity to a takeaway and childhood obesity.

This study is not without limitations which might explain the lack of associations identified. Firstly, the data are cross sectional, therefore limiting our ability to draw causal inference. As with many other studies, information on where people actually shopped or ate was not known. In addition the 2km buffer to define the three environments was an arbitrary distance. Although they provided a measure of local purchasing potential, we had no information on where the children actually ate or purchased food. It is likely that our neighbourhoods do not represent the locations used to actually buy food. New generation studies are beginning to show most people do not shop for food in their immediate neighbourhoods and neighbourhoods are likely to vary from person to person [43], although this data is based on adults. There was little in the literature to guide our decision in relation to the size of the buffer, our definition was selected as this was considered a plausible walking distance and it is in line with some published research [9,17]. The limitations of using arbitrary definitions of a neighbourhood are not unique to this study and have been discussed previously [45]. Although we considered the food environment around schools we did not control for food available within the school premises and if pupils left the school during meal times. In addition all pupils were in the first year at secondary school and so had only been exposed to the school environment for a maximum of 1 year and we did not have information on how long they had lived at their home address. As in previous studies the outlet classification system that we used did not consider the heterogeneity of food offerings in general categories of food outlets. This simple classification system is likely to be a contributing factor to the equivocal results observed in the literature and may also contribute to the lack of associations observed in this study. There are also limitations of using the shortest straight line distance to capture the commuting route. No information was available on the actual route each child took to school however, the buffer around this line was intended to capture all possible routes between a child’s home and school. Although this method has been employed previously [30] GIS modelled routes may overestimate exposure to food outlets compared to GPS measured exposure [46]. Finally, temporal mismatch, which arises when data from different time points are used in cross sectional research, was inevitable. The RADs data was collected between 2005 – 2007 which pre-dates the food environment database. This is a common consideration is this type of research [47].

Finally, the classifications of food outlets (supermarkets, retail and takeaways) was based on the database held by Leeds City Council, this classification may in part explain the null results reported here when compared to studies that have used different classifications.

## Conclusions

The current evidence lends tentative support to the hypothesis that food availability bares an independent relationship to obesity in children. While consumption of fast food may be associated with obesity, this study provides little support for the notion that exposure to fast food and other food outlets in the home, school and commuting neighbourhoods increase the risk of obesity in children. If exposure to food stores bares no relationship to obesity in children, as suggested by the findings here, it is worth asking whether targeting limited food availability represents a promising childhood obesity strategy. This analysis does not imply that fast food restaurants are healthy or that they should be excluded from public health recommendations. However, these results suggest that policy makers should approach policies designed to limit fast food with caution, for example the current zoning laws being proposed.

## Abbreviations

RADS: Rugby League and Athletics Development Scheme
BMI: Body mass index
sBMI: Standardised BMI
SES: Socio economic status
IDACI: Income deprivation affecting children index
